# Association of Shift Work, Health Behaviors, and Socioeconomic Status with Diabesity in over 53,000 Spanish Employees

**DOI:** 10.3390/jcm14175969

**Published:** 2025-08-23

**Authors:** Javier Tosoratto, Pedro Juan Tárraga López, Ángel Arturo López-González, Joan Obrador de Hevia, Carla Busquets-Cortés, José Ignacio Ramírez-Manent

**Affiliations:** 1 Investigation Group ADEMA SALUD, University Institute for Research in Health Sciences (IUNICS), 07010 Palma, Spainjignacioramirez@telefonica.net (J.I.R.-M.); 2 Faculty of Medicine, UCLM (University of Castilla La Mancha), 02008 Albacete, Spain; 3 SESCAM (Health Service of Castilla La Mancha), 02008 Albacete, Spain; 4 Faculty of Dentistry, University School ADEMA, 07010 Palma, Spain; 5 Institut d’Investigació Sanitària de les Illes Balears (IDISBA), Balearic Islands Health Research Institute Foundation, 07010 Palma, Spain; 6 Balearic Islands Health Service, 07010 Palma, Spain

**Keywords:** diabesity, shift work, BMI, CUN-BAE, occupational health, Mediterranean diet, insulin resistance, metabolic syndrome

## Abstract

**Background**: Diabesity, the coexistence of obesity and type 2 diabetes, is a major public health concern. Shift work and unhealthy lifestyle behaviors may exacerbate its prevalence, particularly in working populations. **Objective**: This study aims to evaluate the association between sociodemographic characteristics, health behaviors, and shift work and the prevalence of diabesity, using both BMI and the CUN-BAE estimator, in a large cohort of Spanish workers. **Methods**: This cross-sectional study included 53,053 workers (59.8% men) aged 18–69 years who underwent occupational health examinations. Diabesity was defined as obesity (BMI ≥ 30 kg/m^2^ or high CUN-BAE) plus fasting glucose ≥ 100 mg/dL or prior diagnosis of diabetes. Adherence to the Mediterranean diet was assessed by the MEDAS questionnaire, physical activity by the IPAQ, alcohol intake by standard drink units (UBEs), and socioeconomic class by the CNAE-11 classification. Shift work was defined according to ILO criteria. Logistic regression was used to assess associations, adjusting for potential confounders. **Results**: Shift work was independently associated with increased odds of diabesity both in men and women. Diabesity prevalence was higher when assessed by CUN-BAE compared with BMI. Age, male sex, lower socioeconomic class, physical inactivity, smoking, poor diet adherence, and alcohol intake were all significantly associated with higher risk. The CUN-BAE index showed superior sensitivity in identifying individuals at risk. **Conclusions**: Shift work and unhealthy behaviors are key determinants of diabesity among Spanish workers. The use of adiposity estimators beyond BMI, such as CUN-BAE, should be encouraged in occupational health surveillance. Workplace-targeted interventions are urgently needed to address this growing metabolic burden.

## 1. Introduction

The global burden of type 2 diabetes mellitus (T2DM) continues to escalate progressively, with an estimated 537 million adults affected in 2021—nearly 90–95% of all diabetes cases globally—and is projected to rise in coming decades [1]. In Europe, over 55 million adults live with diabetes in 2023, a figure expected to grow to 64 million by 2030 [2]. Spain exhibits a particularly high burden: recent national cohort and nutritional survey data report an adult obesity prevalence of approximately 21–22%, with overweight prevalence near 39% [3,4]. Among adults aged ≥65 years, both obesity and diabetes are substantially more prevalent [3]. Diabetes affects approximately one in seven Spanish adults, giving Spain one of the highest national prevalences in Europe. In Spain, the nationwide di@bet.es cohort study reported an incidence of diabetes of 11.6 cases per 1000 person-years in adults aged 18–75 during the 2010–2015 period [5].

Obesity, defined as excess adiposity (BMI ≥ 30 kg/m^2^), is a multifactorial disease driven by hormonal, neural, inflammatory, and environmental factors. Affecting over one billion people in 2024, it accounts for one in eight non-communicable disease deaths, mainly via diabetes, cardiovascular disease, certain cancers, and stroke [6]. Pathogenesis involves hypothalamic appetite dysregulation, leptin resistance, gut–brain signaling defects, incretin impairment, and chronic low-grade inflammation, leading to metabolic dysfunction [7].

Type 2 diabetes mellitus is marked by chronic hyperglycemia from insulin resistance and pancreatic β-cell dysfunction, with elevated glucose driving a cycle of glucotoxicity and β-cell decline [8]. Insulin resistance in adipose tissue, liver, and muscle is promoted by excess free fatty acids, pro-inflammatory cytokines, and ectopic fat. Dysglycemia is further aggravated by impaired incretin action, high glucagon, renal sodium retention, and neurohormonal dysregulation [9,10]. T2DM accelerates atherosclerosis and increases microvascular and macrovascular complications, as well as risks of cognitive decline and metabolic dysfunction-associated steatotic liver disease (MASLD) [1,11].

Diabesity refers to the coexistence and interaction of obesity and type 2 diabetes, forming a continuum where excess adiposity is the strongest risk factor for T2DM [12]. This synergy amplifies cardiometabolic risk through obesity-induced insulin resistance, lipotoxicity, β-cell failure, systemic inflammation, and endothelial damage [13]. Proteomic studies reveal biomarkers of adipocyte stress and lipotoxicity underlying its pathophysiology [14]. Clinically, diabesity greatly increases risks of cardiovascular disease, MASLD, renal impairment, obstructive sleep apnea, hypertension, dyslipidemia, and multiple obesity-related cancers [12].

Diabesity, the coexistence of obesity and type 2 diabetes, is common, with 60–80% of T2DM cases in European-origin populations linked to excess adiposity [6,15]. In Spain, regional data are scarce, but one large working population study found prevalence between 2.6% and 5.8%, varying by adiposity measure used (e.g., BMI vs. CUN-BAE vs. others) [16]. The Clínica Universidad de Navarra-Body Adiposity Estimator (CUN-BAE), a validated index that estimates body-fat percentage based on age, sex, and BMI, has demonstrated superior predictive capacity for metabolic risk compared with BMI alone. Global projections anticipate continued increases in both obesity and T2DM, with diabesity representing an expanding public health challenge over the next two decades [1,2].

CUN-BAE, a body adiposity estimator, outperforms BMI, waist circumference, and waist-to-height ratio in predicting diabetes and metabolic syndrome, particularly in Spanish populations [17,18]. A large Chinese cohort confirmed its stronger hazard ratios for incident T2DM [19], supporting the use of adiposity measures beyond BMI for early detection.

Diabesity management strategies include lifestyle changes, pharmacotherapy (e.g., GLP-1 receptor agonists), metabolic surgery, and public health measures [20]. The Mediterranean diet (MD) protects against obesity and T2DM, improving insulin sensitivity and glycemic control [20,21]. Despite global recognition and strong evidence, adherence in Spain is moderate to low, influenced by demographics and socioeconomic status [22]. A systematic review of Mediterranean patients with T2DM showed moderate adherence overall, driven by physical activity, BMI, age, and education [23]. The PREDIMED trial demonstrated a 30% reduction in major cardiovascular events and improved diabetes outcomes with a MD plus olive oil or nuts [24]. Integrative dietary interventions remain crucial for diabesity prevention and management [25].

Obesity and type 2 diabetes, frequently coexisting as diabesity, are among the most prevalent and impactful chronic diseases worldwide. Their joint occurrence significantly increases cardiometabolic morbidity and mortality, especially among populations exposed to unhealthy lifestyles and adverse social determinants of health. Shift work, which disrupts circadian rhythms, has been consistently linked to metabolic disturbances. Therefore, understanding the relationship between shift work, socioeconomic status, and lifestyle factors and diabesity is crucial for preventive strategies.

## 2. Materials and Methods

This cross-sectional study was conducted among Spanish workers undergoing mandatory occupational health evaluations between January 2021 and December 2023, carried out by certified occupational health professionals. All data were obtained from routine occupational health examinations conducted across several Spanish regions as part of a standardized health surveillance program. These evaluations were performed by licensed medical professionals, and data were collected electronically and anonymized.

### 2.1. Study Design and Population

**Figure 1:**
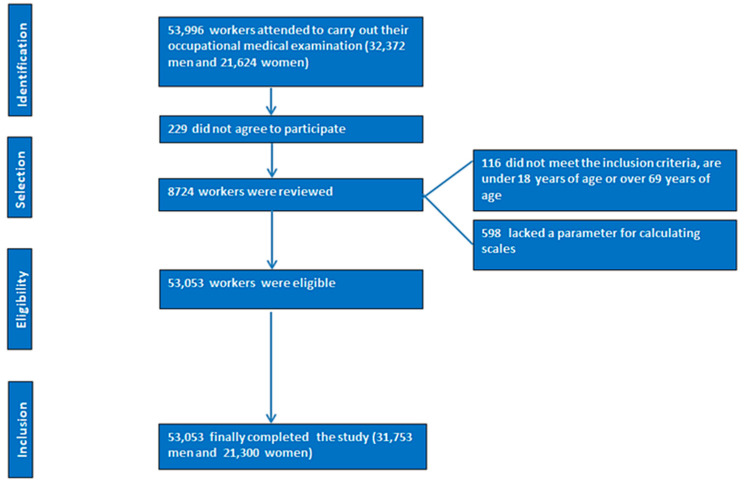
Participant flow diagram for inclusion in the study cohort.

A total of 53,996 individuals (32,372 men and 21,624 women) initially attended medical assessments. After excluding 229 individuals who declined participation, 8724 cases were reviewed for eligibility. This cross-sectional study included Spanish employees who underwent mandatory occupational health examinations between January 2021 and December 2023. Details of participant selection are shown in Figure 1. Workers with incomplete data required for diabesity classification were excluded.

### 2.2. Inclusion and Exclusion Criteria

Inclusion criteria were

- Active workers aged between 18 and 69 years;
- Undergoing a routine occupational medical examination;
- Availability of complete data for calculating anthropometric and metabolic indices (BMI, CUN-BAE, glucose, lipids, etc.).

Exclusion criteria were

- Age under 18 or over 69 years;
- Refusal to participate;
- Incomplete data on any of the variables needed to compute diabesity-related scales.

Inclusion required complete data for calculating anthropometric and metabolic indices, including BMI, CUN-BAE, glucose, and lipids. Exclusion criteria included missing data necessary for diabesity classification or age out of range.

### 2.3. Anthropometric and Clinical Measurements

Body weight and height were measured using standardized procedures, with subjects wearing light clothing and no shoes. BMI was calculated using weight in kilograms divided by the square of height in meters (kg/m^2^). The Clínica Universidad de Navarra-Body Adiposity Estimator (CUN-BAE) was used as an additional indicator of adiposity. Blood pressure, glucose, and lipid profiles (total cholesterol, HDL, LDL, and triglycerides) were obtained through fasting blood samples collected during the medical exam.

### 2.4. Assessment of Lifestyle and Occupational Variables

Physical activity was categorized according to self-reported frequency, duration, and intensity [26], and dietary habits were evaluated through adherence to a Mediterranean diet index based on the frequency of consumption of key food groups [27]. Tobacco consumption was classified as current smoking, and former smokers were those who had abstained from smoking for more than one year or never smoked. A current smoker was defined as someone who had smoked at least 100 cigarettes in their lifetime and currently smoked either daily or occasionally. Alcohol consumption was assessed as the average number of standard drink units (UBEs) per week, following the guidelines of the Spanish Ministry of Health [28].

Socioeconomic class was classified using the CNAE-11 (Clasificación Nacional de Actividades Económicas), following the criteria of the Spanish Society of Epidemiology, stratified into three categories (I: higher, II: intermediate, and III: lower class) [29].

Shift work status was defined according to the International Labour Organization (ILO) criteria, including night shifts, rotating schedules, or work during non-standard hours [30].

### 2.5. Definition of Diabesity

Diabesity was defined as the concurrent presence of obesity (BMI ≥30 kg/m^2^ or adiposity above CUN-BAE thresholds) and impaired fasting glucose (≥100 mg/dL) or a diagnosis of type 2 diabetes mellitus. Prevalence and associations were analyzed according to sex, age groups, social class, education, physical activity, Mediterranean diet adherence, smoking, alcohol consumption, and shift work [31].

### 2.6. Statistical Analysis

All analyses were performed using SPSS version 29.0 (IBM Corp., Armonk, NY, USA). Descriptive statistics were used to summarize sociodemographic, clinical, and lifestyle characteristics. Continuous variables are presented as means and standard deviations (SDs), while categorical variables are reported as percentages. Normality of continuous variables was assessed using the Shapiro–Wilk test. Since variables showed approximately normal distributions, parametric tests such as Student’s t-test and ANOVA were used for comparisons.

Multivariate logistic regression models were conducted to identify factors associated with diabesity, as defined by both BMI and CUN-BAE. Adjusted odds ratios (ORs) with 95% confidence intervals (CIs) were estimated. Variables included in the models were age, sex, smoking status, physical activity, alcohol intake, Mediterranean diet adherence, social class, and shift work. Potential confounders were identified and included in the models. Multicollinearity was assessed using variance inflation factors (VIF), and model fit was evaluated using the Hosmer–Lemeshow test. A *p*-value < 0.05 was considered statistically significant.

## 3. Results

Table 1 presents the baseline characteristics of 53,053 Spanish workers stratified by sex and shift-work status. Overall, male and female shift workers exhibited significantly higher anthropometric (weight and waist circumference), clinical (blood pressure, glucose, and lipid profiles), and lifestyle-related risk factors compared with their non-shift counterparts. Notably, shift workers of both sexes had significantly lower adherence to physical activity and the Mediterranean diet, along with a higher prevalence of smoking and alcohol consumption. Socioeconomic disparities were also evident, with shift workers more frequently belonging to lower educational and social-class categories. These findings underscore the adverse cardiometabolic and lifestyle profiles associated with shift work, which may contribute to elevated diabesity risk in this subgroup.

Table 2 details the prevalence of diabesity among male workers using both BMI- and CUN-BAE-based definitions. Diabesity prevalence increased with age and was consistently higher among shift workers across all age strata. Moreover, lower educational attainment and belonging to social class III were associated with increased diabesity, with a more pronounced prevalence when assessed via CUN-BAE. Importantly, unhealthy behaviors—including smoking, physical inactivity, low adherence to the Mediterranean diet, and alcohol consumption—were linked to significantly higher diabesity rates. These results reinforce the sensitivity of CUN-BAE in detecting body-fat-related metabolic risk and highlight the importance of addressing modifiable behaviors in male shift workers.

Table 3 mirrors the analysis in Table 2, focusing on female workers. Although overall diabesity prevalence was lower than in men, a similar pattern was observed: higher rates among older age groups, shift workers, those with lower socioeconomic status, and individuals with unhealthy lifestyle behaviors. Again, the CUN-BAE estimator identified substantially more cases of diabesity than BMI alone, especially in older women and those with limited physical activity or low Mediterranean diet adherence. These findings suggest sex-specific considerations in diabesity screening and intervention, emphasizing the utility of adiposity estimators beyond BMI in occupational health assessments.

Table 4 displays the results of multivariate logistic regression models assessing the association between sociodemographic, educational, behavioral, and occupational variables and the odds of having diabesity, using both BMI and CUN-BAE definitions. Male sex, older age, lower social class and educational attainment, smoking, physical inactivity, poor adherence to the Mediterranean diet, alcohol consumption, and shift work were all independently associated with higher odds of diabesity. Notably, shift work was associated with a twofold increase in risk (OR = 2.22 for BMI-based and OR = 2.62 for CUN-BAE-based diabesity). The strongest predictors across models were physical inactivity and non-adherence to the Mediterranean diet. The higher odds ratios observed with CUN-BAE-based models further support its superior discriminatory power in identifying individuals with diabesity risk, particularly in the context of workplace health surveillance.

## 4. Discussion

This large-scale, cross-sectional study among 53,053 Spanish workers revealed that shift work is significantly associated with a higher prevalence of diabesity, whether defined by BMI ≥ 30 kg/m^2^ or via the CUN-BAE adiposity estimator. Shift workers across both sexes exhibited worse anthropometric, clinical, and lifestyle profiles—including higher rates of smoking, alcohol consumption, physical inactivity, and poor Mediterranean diet adherence—and had consistently higher odds of diabesity after adjustment for sociodemographic and behavioral confounders. These findings are consistent with prior evidence showing elevated obesity and insulin resistance markers among shift workers in Spain [32] and extend them to combined diabesity outcomes.

Our results complement national data showing diabesity prevalence in working populations ranging from 2.6% (BMI) to 5.8% (other formulas) and its strong associations with age, male sex, and low socioeconomic status [33]. The independent contribution of shift work to diabesity risk underscores the role of occupational circadian disruption as a modifiable determinant of metabolic dysfunction, even when accounting for known confounders, such as lifestyle factors and social class. Meta-analytic data confirm an increased risk of type 2 diabetes incidence by approximately 30% (HR = 1.30, 95% CI 1.18–1.43) in night shift workers, with stronger effects in female workers and longer duration exposures [34].

Furthermore, the CUN-BAE estimator proved more sensitive than BMI in detecting diabesity risk associated with shift work. Prior studies found that each two-unit increase in CUN-BAE correlated with a 46% increase in incident T2DM risk (95% CI 33–62%) in large cohorts [18]. In our cohort, CUN-BAE-based diabesity prevalence was systematically higher than BMI-based estimates, especially among shift workers, older adults, women, and lower-social-class groups, supporting its use as a superior screening tool in occupational health.

Our findings align with research observing higher obesity prevalence among shift workers across multiple adiposity indices (BMI, WtHR, CUN-BAE, and METS VF) and confirm sex differences—with men showing stronger associations between shift work and obesity, although women were also affected via visceral adiposity measures [35]. This pattern underscores the consistency of our observed associations across multiple methodologies.

International meta-analyses have consistently linked shift work to increased risk of obesity and T2DM, with relative risks typically ranging from 1.1 to 1.6 depending on shift duration, type, and population characteristics [36,37]. Danish nurse cohorts and Atlantic Path studies reflect similar associations even after adjusting for BMI [38]. Our results thereby provide context-specific evidence in a large Spanish working cohort, confirming the occupational health relevance of shift-work-induced metabolic risk.

It is worth noting that even in individuals without diabetes, higher levels of glycated hemoglobin (HbA1c) have been associated with subclinical cardiovascular damage and increased risk of atherosclerosis. This supports the hypothesis that early glycemic dysregulation may contribute to cardiovascular burden even before diabetes is clinically evident [39].

Moreover, although metabolic dysfunction–associated steatotic liver disease (MASLD) was not a primary outcome, recent studies indicate that shift work also independently predicts elevated MASLD risk using non-invasive indices, particularly among women and blue-collar workers [40,41,42]. This suggests that the metabolic burden of diabesity in shift workers may extend to hepatic consequences, reinforcing the need for broad cardiometabolic surveillance.

Longitudinal cohort studies are essential to establish temporal relationships between shift work and the onset of diabesity. Incorporating baseline metabolic profiles and follow-up data will help clarify causality, enabling researchers to distinguish whether metabolic disturbances precede or result from shift work exposure. Objective measurement of circadian disruption—through wearable-based sleep tracking, melatonin assays, and detailed characterization of shift duration and rotation patterns—could substantially refine exposure assessment. Such precise quantification would also facilitate exploration of the mechanistic pathways involved, such as the chronodisruption–inflammation–insulin resistance axis, thereby deepening understanding of how altered biological rhythms contribute to metabolic deterioration [43].

In parallel, integrating genetic and multi-omics data, including proteomic and metabolomic biomarkers, holds promise for identifying molecular pathways that link adiposity, circadian misalignment, and metabolic dysfunction in shift workers [44]. Interventional studies targeting modifiable factors—such as diet, physical activity, sleep hygiene, and optimized scheduling—could offer actionable strategies to mitigate risk. For instance, testing whether Mediterranean diet promotion in shift workers reduces diabesity incidence may provide robust, context-specific evidence for workplace health initiatives. Moreover, tailoring prevention programs by sex, age, and socioeconomic status may enhance their effectiveness, especially given the higher vulnerability observed in men and those from lower social strata. Finally, broadening the scope of outcomes to include liver health (e.g., MASLD), cardiovascular endpoints, sleep disorders, and even cancer risk would deliver a more comprehensive picture of the health consequences arising from the intersection of shift work and diabesity, ultimately guiding both clinical practice and occupational health policy.

This study offers several noteworthy contributions. Our findings demonstrate that shift work operates as an independent risk factor for diabesity, while concurrently revealing the superior sensitivity of the CUN-BAE index over BMI in detecting excess adiposity in this occupational context. By elucidating key lifestyle mediators, including poor dietary quality, insufficient physical activity, smoking, and alcohol consumption, this study highlights partially modifiable pathways through which shift work may contribute to metabolic deterioration, thereby underscoring the potential of targeted behavioral interventions. Furthermore, the identification of disproportionate risk among men, older employees, and individuals in lower socioeconomic strata emphasizes the need for sex- and social-class–specific preventive strategies. These results carry important occupational-health-policy implications, supporting the inclusion of work schedule management as a core component of workplace programs aimed at reducing the burden of metabolic diseases. Finally, the observed association between diabesity in shift workers and indices indicative of metabolic dysfunction–associated steatotic liver disease (MASLD) expands the scope of concern, suggesting a shared pathogenic axis that warrants further investigation into hepatic outcomes within this high-risk population.

## 5. Strengths

- Large, representative occupational cohort: Inclusion of 53,053 workers across diverse sectors and geographic regions yields high statistical power and external validity for employed Spanish adults.
- Use of multiple adiposity definitions: Comparing BMI and CUN-BAE allows for demonstration of differential sensitivity in diabesity detection and reinforces the utility of adiposity estimators beyond traditional measures.
- Rigorous adjustment for confounders: Multivariate models controlled for important sociodemographic (age, sex, and social class), behavioral (diet, activity, smoking, and alcohol), and occupational (shift work) variables, reducing residual confounding.
- Sex-stratified analyses: Sex-stratified analyses permitted the elucidation of sex differences in diabesity prevalence and shift-work associations.
- Validated measurement instruments: MEDAS, IPAQ, UBEs, and CNAE-11 classifications ensured standardized, comparable data collection across participants.

## 6. Limitations

- Cross-sectional design: The cross-sectional design prevents causal inference and temporal relationships, limiting the ability to establish that shift work precedes diabesity onset. Reverse causation or residual confounding remains possible.
- Self-reported behavioral data: Measures such as diet adherence (MEDAS), physical activity (IPAQ), and alcohol consumption (UBEs) rely on self-report and may be subject to recall or social-desirability bias.
- Lack of biochemical confirmation of diabetes: Glycemic status was defined based on fasting glucose ≥100 mg/dL or prior diagnosis; no oral glucose tolerance testing or HbA1c was systematically performed.
- Absence of objective sleep/circadian data: Quantitative measures such as shift duration, chronotype, objectively assessed sleep quality, and melatonin profiles were not included; occupational exposure was broadly defined via ILO criteria.
- Potential selection bias: Participants recruited during employer-mandated health examinations may not represent unemployed individuals or those not covered by occupational screening, limiting generalizability beyond the selected working population.

## 7. Conclusions

This large-scale cross-sectional study confirms that shift work is independently associated with an increased risk of diabesity, particularly when adiposity is assessed through the CUN-BAE estimator rather than BMI alone. The analysis demonstrates that sex, age, social class, and lifestyle behaviors such as smoking, low physical activity, poor adherence to the Mediterranean diet, and alcohol consumption are significant modifiers of this risk.

The use of the CUN-BAE index, which accounts for body-fat percentage more accurately than BMI, revealed a notably higher prevalence of diabesity, especially among older adults, women, and shift workers. This highlights the limitations of BMI-based approaches and supports the integration of more sensitive adiposity estimators into occupational health surveillance.

The association between non-standard work schedules and metabolic dysfunction is increasingly evident and supports the need to monitor and mitigate the impact of shift work on employee health. These findings underscore the importance of implementing workplace-based interventions, including nutritional counseling, physical-activity programs, and shift redesign to reduce circadian misalignment.

Public health strategies should consider the role of the workplace not only as a setting for risk but also for intervention. Screening programs using CUN-BAE, coupled with promotion of healthy behaviors among shift workers and disadvantaged groups, may be key to reducing the burden of diabesity and its associated complications.

Future prospective and interventional studies are needed to confirm these associations over time and to guide evidence-based occupational health policies. This study also sets the foundation for multifactorial prevention strategies targeting metabolic health inequalities across different occupational and demographic profiles.

## Figures and Tables

**Table 1:** Sociodemographic, clinical, and lifestyle characteristics of workers by shift status and sex.

	Non-Shift Work	Shift Work	*p*-Value	Non-Shift Work	Shift Work	*p*-Value
	Men n = 14,226	Men n = 17,527		Women n = 10,019	Women n = 11,281	
	Mean (SD)	Mean (SD)		Mean (SD)	Mean (SD)	
Age (years)	41.2 (10.9)	41.3 (10.5)	0.089	40.0 (10.5)	40.2 (10.3)	0.199
Height (cm)	173.8 (7.1)	173.7 (7.1)	0.219	161.0 (6.6)	161.2 (6.6)	0.015
Weight (kg)	81.5 (14.6)	84.5 (14.4)	<0.001	63.6 (12.8)	68.6 (12.8)	<0.001
Waist (cm)	89.5 (10.5)	90.8 (10.2)	<0.001	74.7 (9.7)	77.6 (10.9)	<0.001
Systolic BP (mmHg)	125.3 (15.7)	126.9 (16.0)	<0.001	114.8 (15.5)	116.1 (15.6)	<0.001
Diastolic BP (mmHg)	75.9 (10.7)	77.2 (11.0)	<0.001	70.3 (10.6)	71.6 (10.8)	<0.001
Total cholesterol (mg(dL)	197.3 (38.4)	201.2 (38.6)	<0.001	192.3 (36.6)	196.9 (37.3)	<0.001
HDL cholesterol (mg/dL)	50.4 (7.8)	49.7 (7.7)	<0.001	55.0 (9.1)	54.5 (9.2)	<0.001
LDL cholesterol (mg/dL)	120.9 (37.3)	123.8 (37.6)	<0.001	119.6 (36.9)	123.5 (37.5)	<0.001
Triglycerides (mmHg)	129.3 (93.7)	136.8 (95.5)	<0.001	87.5 (46.8)	93.6 (51.7)	<0.001
Glucose (mg/dL)	91.9 (26.4)	93.3 (26.4)	<0.001	86.6 (19.0)	87.8 (17.6)	<0.001
	**%**	**%**	***p*** **-Value**	**%**	**%**	***p*** **-Value**
18–29 years	16.4	13.8	<0.001	18.6	17.5	0.135
30–39 years	29.3	29.8		31.0	31.3	
40–49 years	29.0	31.3		29.6	30.6	
50–59 years	20.9	20.9		17.9	17.5	
60–69 years	4.4	4.2		2.9	3.1	
Social class I	6.8	8.2	<0.001	11.6	14.6	<0.001
Social class II	20.7	26.6		27.6	37.0	
Social class III	72.5	65.2		60.8	48.4	
Elementary school	69.5	63.8	<0.001	53.7	43.2	<0.001
High school	24.4	28.9		36.2	44.2	
University	6.1	7.3		10.1	12.6	
Non-smokers	67.9	66.0	<0.001	66.3	69.1	<0.001
Smokers	32.1	34.0		33.7	30.9	
Non-physical activity	55.2	67.9	<0.001	40.8	60.7	<0.001
Yes physical activity	44.8	32.1		59.2	39.3	
Non-Mediterranean diet	58.2	71.5		42.0	63.1	
Yes Mediterranean diet	41.8	28.5		58.0	36.9	
Non-alcohol consumption	70.4	63.2	<0.001	85.3	83.5	<0.001
Yes alcohol consumption	29.6	36.8		14.7	16.5	

**Table 2:** Prevalence of diabesity according to BMI and CUN-BAE by age, social class, education, and lifestyle in male workers, stratified by shift status.

		Non-Shift Work			Shift Work	
		Diabesity BMI	Diabesity CUN-BAE		Diabesity BMI	Diabesity CUN-BAE
Men	n	%	%	n	%	%
18–29 years	2329	0.3	0.6	2425	0.5	0.8
30–39 years	4174	1.0	1.7	5228	1.3	1.9
40–49 years	4130	2.4	4.6	5477	3.1	5.8
50–59 years	2972	6.2	11.1	3666	7.4	14.1
60–69 years	621	9.2	20.4	731	10.1	22.2
Social class I	972	2.1	3.6	1438	2.7	5.9
Social class II	2942	2.4	3.9	4669	3.2	6.1
Social class III	10,312	2.9	5.8	11,420	3.5	6.3
Elementary school	9874	2.9	5.7	11,169	3.6	6.4
High school	3478	2.5	4.0	5070	3.3	6.2
University	874	2.0	3.5	1288	2.6	5.7
Non-smokers	9656	2.8	4.4	11,567	3.5	5.5
Smokers	4570	2.9	5.4	5960	3.7	6.6
Non-physical activity	7851	4.1	6.9	11,899	5.9	10.2
Yes physical activity	6375	0.4	1.2	5628	0.7	1.4
Non-Mediterranean diet	8275	3.9	6.0	12,536	5.5	8.8
Yes Mediterranean diet	5951	0.8	1.9	4991	1.2	2.6
Non-alcohol consumption	8996	1.1	5.5	12,332	1.4	7.9
Yes alcohol consumption	5230	3.5	2.2	5195	4.8	3.3

BMI: Body mass index. CUN-BAE: Clinica Universitaria de Navarra-Body Adiposity Estimator.

**Table 3:** Prevalence of diabesity according to BMI and CUN-BAE by age, social class, education, and lifestyle in female workers, stratified by shift status.

		Non-Shift Work			Shift Work	
		Diabesity BMI	Diabesity CUN-BAE		Diabesity BMI	Diabesity CUN-BAE
Women	n	%	%	n	%	%
18–29 years	1869	0.2	0.1	1975	0.4	0.2
30–39 years	3103	0.6	0.8	3530	0.8	0.9
40–49 years	2965	1.2	2.4	3450	1.5	2.7
50–59 years	1791	3.1	6.6	1974	4.3	7.1
60–69 years	291	4	10.5	352	5.8	13.4
Social class I	1164	0.2	0.9	1644	0.3	1.1
Social class II	2763	1.0	1.4	4175	1.3	2.0
Social class III	6092	2.0	3.4	5462	2.5	4.0
Elementary school	5377	2.1	3.3	4871	2.4	3.9
High school	3628	1.0	1.6	4984	1.3	2.2
University	1014	0.9	0.7	1426	0.3	1.0
Non-smokers	6638	1.3	2.8	7794	1.6	3.4
Smokers	3381	1.4	3.2	3487	1.8	3.7
Non-physical activity	4090	2.5	2.3	6842	4.0	4.0
Yes physical activity	5929	0.1	0.3	4439	0.3	0.4
Non-Mediterranean diet	4206	2.3	2.0	7115	3.6	3.5
Yes Mediterranean diet	5813	0.4	0.7	4166	0.8	0.9
Non-alcohol consumption	8361	0.6	1.9	9619	1.1	3.0
Yes alcohol consumption	1658	2.0	0.9	1662	3.1	1.3

**Table 4:** Multivariate logistic regression analysis of factors associated with diabesity according to BMI and CUN-BAE.

	Diabesity BMI	*p*-Value	Diabesity CUN-BAE	*p*-Value
	OR (95% CI)		OR (95% CI)	
Women	1		1	
Men	1.59 (1.49–1.70)	<0.001	1.21 (1.17–1.26)	<0.001
18–29 years	1		1	
30–39 years	1.21 (1.17–1.26)	<0.001	1.51 (1.39–1.63)	<0.001
40–49 years	1.89 (1.73–2.05)	<0.001	2.77 (2.35–3.20)	<0.001
50–59 years	3.07 (2.65–3.50)	<0.001	4.86 (3.97–5.76)	<0.001
60–69 years	7.49 (6.08–8.90)	<0.001	8.95 (7.35–10.55)	<0.001
Social class I	1		1	
Social class II	1.20 (1.15–1.26)	<0.001	1.65 (1.42–1.88)	<0.001
Social class III	1.58 (1.46–1.70)	<0.001	2.12 (1.63–2.62)	<0.001
University	1		1	
High school	1.23 (1.16–1.31)	<0.001	1.60 (1.40–1.81)	<0.001
Elementary school	1.57 (1.45–1.69)	<0.001	2.13 (1.65–2.62)	<0.001
Non-smokers	1		1	
Smokers	1.28 (1.20–1.36)	<0.001	1.33 (1.26–1.41)	<0.001
Yes physical activity	1		1	
Non-physical activity	12.62 (9.95–15.30)	<0.001	9.87 (8.08–11.67)	<0.001
Yes Mediterranean diet	1		1	
Non-Mediterranean diet	6.85 (5.12–8.59)	<0.001	5.21 (4.15–6.27)	<0.001
Non-alcohol consumption	1		1	
Yes alcohol consumption	4.23 (3.18–5.29)	<0.001	5.12 (4.09–5.16)	<0.001
Non-shift work	1		1	
Yes shift work	2.22 (1.89–2.56)	<0.001	2.62 (2.03–3.22)	<0.001

BMI: Body mass index. CUN-BAE: Clinica Universitaria de Navarra-Body Adiposity Estimator. OR: Odds ratio.

## Data Availability

The data collected for this study are stored in a secure institutional database that complies with all legal and technical requirements at ADEMA University School. The appointed Data Protection Officer is Ángel Arturo López González.

## Abbreviations

The following abbreviations are used in this manuscript:

AJHS	Academic Journal of Health Sciences
AVAI	Abdominal Volume Adiposity Index
BAI	Body Adiposity Index
BMI	Body Mass Index
BP	Blood Pressure
CEI-IB	Ethics and Research Committee of the Balearic Islands (Comité de Ética de la Investigación de las Islas Baleares)
CI	Confidence Interval
CNAE-11	National Classification of Economic Activities, 2011 (Spain)
CUN-BAE	Clínica Universidad de Navarra-Body Adiposity Estimator
DAI	Dysfunctional Adiposity Index
DM2/T2DM	Type 2 Diabetes Mellitus
ER	Endoplasmic Reticulum (contextual to ER stress)
GDPR	General Data Protection Regulation
GLP-1	Glucagon-Like Peptide-1
HDL	High-Density Lipoprotein
HR	Hazard Ratio
IDF	International Diabetes Federation
ILO	International Labour Organization
IPAQ	International Physical Activity Questionnaire
IAPP	Islet Amyloid Polypeptide
IR	Insulin Resistance
JCM	Journal of Clinical Medicine
LDL	Low-Density Lipoprotein
MAFLD	Metabolic Dysfunction-Associated Fatty Liver Disease (previously NAFLD)
MASLD	Metabolic Dysfunction-Associated Steatotic Liver Disease
MEDAS	Mediterranean Diet Adherence Screener
METS-IR	Metabolic Score for Insulin Resistance
NAFLD	Non-Alcoholic Fatty Liver Disease
OR	Odds Ratio
PMCID	PubMed Central Identifier
PMID	PubMed Identifier
Q1	Quartile 1 (Top 25% in Journal Rankings)
SD	Standard Deviation
SPISE-IR	Single-Point Insulin Sensitivity Estimator for IR
T2DM	Type 2 Diabetes Mellitus
TNF-α	Tumor Necrosis Factor Alpha
UBEs	Standard Drink Units (Spanish: Unidades de Bebida Estándar)
VAI	Visceral Adiposity Index
VIF	Variance Inflation Factor
WC	Waist Circumference
WHtR	Waist-to-Height Ratio
